# Improved TV-CS Approaches for Inverse Scattering Problem

**DOI:** 10.1155/2015/262985

**Published:** 2015-10-01

**Authors:** M. T. Bevacqua, L. Di Donato

**Affiliations:** ^1^University Mediterranea of Reggio Calabria, Via Graziella, Località Feo di Vito, 89124 Reggio di Calabria, Italy; ^2^University of Catania, Viale Andrea Doria 6, 95126 Catania, Italy

## Abstract

Total Variation and Compressive Sensing (TV-CS) techniques represent a very attractive approach to inverse scattering problems. In fact, if the unknown is piecewise constant and so has a sparse gradient, TV-CS approaches allow us to achieve optimal reconstructions, reducing considerably the number of measurements and enforcing the sparsity on the gradient of the sought unknowns. In this paper, we introduce two different techniques based on TV-CS that exploit in a different manner the concept of gradient in order to improve the solution of the inverse scattering problems obtained by TV-CS approach. Numerical examples are addressed to show the effectiveness of the method.

## 1. Introduction

The capability of solving in a fast and accurate fashion inverse scattering problems has an enormous interest in fields as different as biomedical imaging, nondestructive evaluation, and subsurface sensing. In all these applications, including the cases concerned with the use of radar or radar-like sensor for subsurface imaging and through the wall imaging, it makes sense to look for methods which allow to reduce as much as possible the number of measurements/sensors while still achieving accurate reconstructions. In this respect, the Compressive Sensing theory (CS) [1, 2] may bring enormous advantages.

In fact, as long as the sought function is known to be sparse or compressible in a given basis, namely it is represented in an exact or anyway accurate fashion through a limited number of nonzero coefficients, the number of measurements actually needed for an accurate reconstruction can be much less than the overall number of unknowns and, moreover, it is possible to obtain nearly optimal reconstructions, as well as a kind of “superresolution” [1, 2].

As well known, the inverse scattering problem, which is a possible framework for quantitative GPR and through the wall imaging, amounts to recover the geometry and the electromagnetic properties of unknown scattering objects, starting from the knowledge of the incident fields and the measurement of the corresponding scattered fields. Unfortunately, the problem is both ill-posed and nonlinear [3], which implies that formidable efforts have to be done to pursue reliable and accurate solutions.

Very many different approaches exist to tackle such a problem, ranging from qualitative methods [4], which simply try to recover information such as presence, location, and possibly shape of unknown targets, to quantitative inversion, for instance [5, 6], which aim to recover the electromagnetic characteristics as well. With respect to the latter, they range from linear to nonlinear approaches, for instance [7, 8], which face the mathematical problem in its full complexity.

The Compressive Sensing theory is well developed for the case of linear problem and, as a result, it is usually used jointly with simplified models, such as the Born or Rytov approximations [7]. Both these linear approximations suffer from several limitations induced by the adopted approximated model. Recently, a new linear approximation has been introduced [9] which outperforms the usual Born approximation and succeeds the latter in imaging nonweak targets [10], thus allowing to significantly enlarge the range of applicability of the CS [11] for inverse scattering problems. In [11], the Compressive Sensing is also used in conjunction with the Total Variation approach [12, 13], which allows to image extended targets, which are nonsparse in the commonly used pixel based representation. In fact, TV is widely used as regularizer especially when the unknown signal is piecewise constant; that is, it has a sparse gradient of the contrast function.

In the following, we consider the joint exploitation of CS and TV approach and try to generalize this latter to improve the reconstruction of objects with discontinuities having different orientation and shape.

The paper is organized as follows. In Section 2, the approximation exploited to linearize the inverse scattering problem is introduced. In Section 3, two different TV-CS based approaches are presented. Finally, in Section 4, a numerical analysis with simulated data is reported to assess the performances of the proposed strategies. Conclusions follow. Throughout the paper, the canonical 2D electromagnetic scalar problem is considered. The exp⁡(*jωt*) time harmonic factor is assumed and dropped.

## 2. Inverse Scattering Problem and the Adopted Linear Approximation

For the sake of simplicity, let us assume that the investigated domain *D* is embedded in a background medium of known complex permittivity *ε*_*b*_ and contains one or more unknown dielectric scatterers with support Σ and complex permittivity *ε*. According to [14], only a limited number of scattering experiments carry all the essential information available for profile inversion. As a consequence, *V* plane waves impinging on *D* from several incident directions evenly spaced in angle are considered and *M* different receivers in the far-field of *D* are located in order to observe the corresponding scattered fields. By assuming the TM polarization, the scalar equations for the generic *v-*illumination are expressed in vector-matrix form as

(1) Esv=A_eEtvχ,

(2) Etv=Eiv+A_iEtvχ,

wherein **χ** is the unknown contrast function and **E**_*s*_^(*v*)^, **E**_*t*_^(*v*)^, and **E**_*i*_^(*v*)^ are, respectively, the vectors which contain the *M* measurements of the scattered field and the values of the total and incident electric field in *D*, respectively. The matrices ${\underline{A}}_{e}$ and ${\underline{A}}_{i}$ are the discretized version of the radiation operators relating the product **E**_*t*_^(*v*)^ **χ** to the scattered field in the observation domain and in the investigation domain, respectively.

As can be seen in (1) and (2), the problem is nonlinear, because of the presence of the term **E**_*t*_^(*v*)^ **χ**. In order to apply the CS to the inverse scattering problem, a new recently introduced linear approximation is considered [9]. The basic idea, which gives rise to this powerful tool, whose range of validity goes beyond the usual Born approximations [10], is the following.

For a fixed contrast function, the scattered field is linearly related to the incident fields. Hence, a linear superposition of the *V* incident fields, adopted to probe *D*, gives rise to a scattered field which is nothing but the same linear superposition of the corresponding measured scattered fields. Such simple reasoning suggests that new “virtual” scattering experiments [9, 11, 15, 16] can be possibly devised (without any need of additional measurements) by simply combining the results of the originally performed scattering experiments. A possible way to design these new experiments, which do not carry any additional information, is to consider the far-field equation, that is, the basic equation of the well-known linear sampling method [4], which allows enforcing, at least approximately, a peculiar spatial distribution of the total field inside *D*.

In particular, one is able, for different “pivot points” located inside the scatterer, to realize virtual scattering experiments wherein the internal fields are focused around the pivot points (see [9, 15, 16]) and hence are foreseeable in an accurate fashion. Such a circumstance allows then (replacing the original experiments with the virtual ones) to deal with a linearization of the scattering equations. In fact (1) can be recast into a linear one, which reads

(3) $$\begin{array}{c} E_{s}^{\left(v\right)}={\underline{A}}_{e}E_{t}^{\left(v\right)}\chi , \end{array}$$

where *E*_*s*_^(*v*)^ and *E*_*t*_^(*v*)^ represent the scattered field data recombined by means of the “design equation,” that is, the LSM equation, and the approximated total field in *D* which arise in the virtual scattering experiments, respectively [9].

## 3. Improved TV-CS Based Inversion Approaches

Let us consider a reference system *Oxy* with the origin in the center of *D* and let us suppose that the inverse scattering problem has been linearized by means of (3). It is known that Compressive Sensing theory provides the tools for reconstructing sparse signals from (highly) incomplete sets of measurements through a constrained *ℓ*_1_ minimization. In a number of cases including nondestructive testing, subsurface sensing, geophysical and biomedical scenarios, it is reasonably to assume that the unknown contrast function has sparse or nearly sparse gradients. As a result, it makes sense to pursue a reconstruction by means of a total variation minimization [12, 13]. Accordingly, the inverse scattering problem can be solved by means of a total variation minimization:

(4) min⁡χ Dxχl1+Dyχl1subject  to Aχ−bl2≤δ,

where **χ** is the *N*-dimensional unknown function of the problem with *N* the number of the pixels discretizing *D*, **b** is the *T* × 1 data vector, *T* = *M* × *P*, which contains the *M* measured scattered fields arising in the *P* virtual scattering experiments, and $A={\underline{A}}_{e}E_{t}^{\left(v\right)}$ is the *T* × *N* matrix which relates the unknown vector to the data vector and, assuming the usual CS terminology, represents the sensing matrix. Finally, **D**_*x*_ and **D**_*y*_ are the discretized version of the partial derivatives evaluated with respect to the spatial variables *x* and *y*, respectively, that is, the discretized version of the gradient along the coordinate directions. In other words, **D**_*x*_ **χ** and **D**_*y*_ **χ** are the *N* × 1 vectors containing the forward differences [13] of the unknown function **χ**.

In (4), the minimization of the sum of the two norms promotes the search of solutions with sparse gradient, while the constraint enforces the data consistency. In other words, among all solutions, which are consistent with the acquired data, we search the one whose gradient has the minimum *ℓ*_1_-norm. Note that the parameter *δ* depends on the level of required accuracy, on the level of noise on the data and on the introduced model error. Notably, the number *T* of data can be (much) less than the overall number of unknowns *N*, but it has to be sufficiently larger than the number of nonzero elements of **D**_*x*_ **χ** and **D**_*y*_ **χ**.

### 3.1. An Orientation Invariant TV-CS Approach

**Figure 1:**
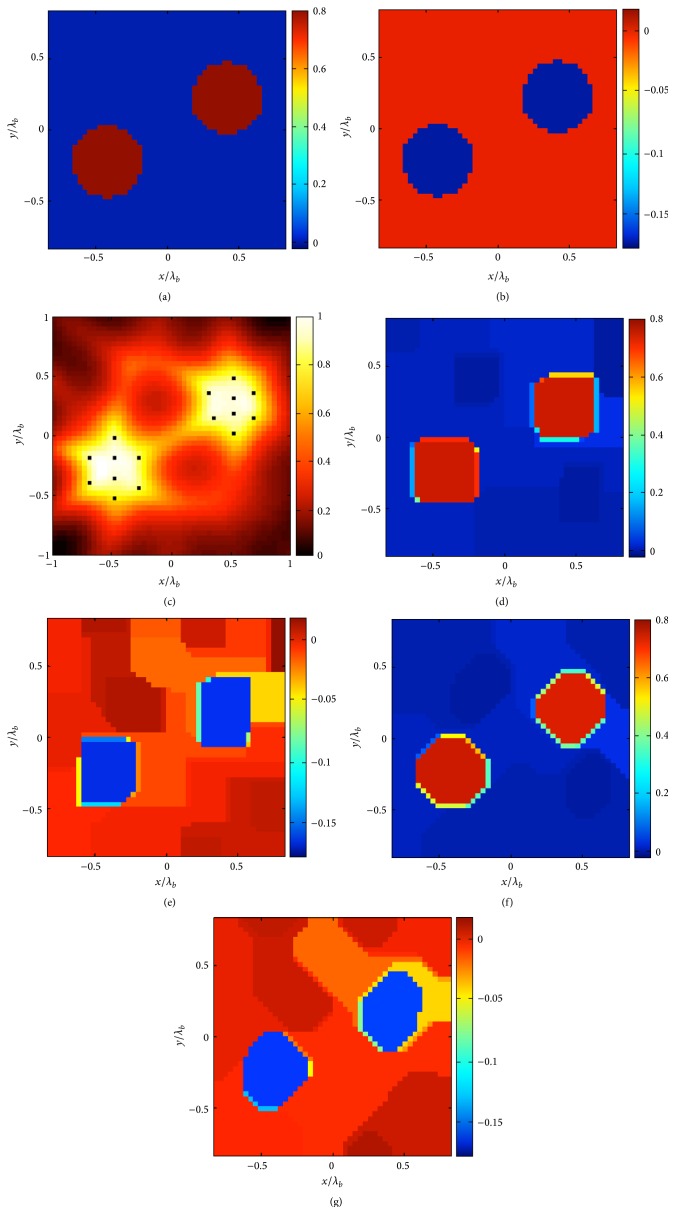
The two-cylinder example. (a) Real and (b) imaginary part of the contrast reference profile. (c) Normalized logarithmic LSM indicator with the selected pivot points superimposed as dots. The retrieved profile by means of the approach (4) (*δ* = 0.2‖**b**‖_*l*_2__ and err = 10%): (d) real and (e) imaginary part. The retrieved profile by means of the approach (5) (*δ* = 0.2‖**b**‖_*l*_2__ and err = 6%): (f) real and (g) imaginary part.

The approach described in (4) is able to identify in a simple fashion the target discontinuities along directions parallel to the coordinate axes. On the other side, since it has two preferential directions, discontinuities having a different orientation are not correctly identified, and the approach provides a kind of “squared” reconstruction of the target (see Figures 1(d) and 1(e)). In order to counteract, at least in a partial fashion, the dependence of the approach with respect to the orientation of the target discontinuities, we propose herein a modified approach.

In particular, we introduce a new objective function which allows to identify additional discontinuities located at + or −45° with respect to the coordinate axes. In such a way, one will have more accurate reconstruction of discontinuities having a generic or even circular shape.

In practice, we consider an additional term defined as the discretized version of the directional derivative **D**_**d**_ evaluated along the directions parallel to *x* = ±*y*. In other words, **D**_**d**_ **χ** is the vector, which contains the forward differences along directions parallel to the principal and secondary diagonals of the matrix of pixels representing the unknown function **χ**. Accordingly, (4) is recast as

(5) min⁡χ Dxχl1+Dyχl1+Ddχl1subject  to Aχ−bl2≤δ.

Roughly speaking, the optimization problem now amounts to looking for a solution whose gradient, evaluated also in the “oblique” directions, has the minimum *ℓ*_1_ norm among all the contrast functions fulfilling (within a given error) the data equation.

### 3.2. A Corner Identifier TV-CS Approach

As a second contribution, we asked ourselves if we can have a still better procedure for profiles where the discontinuities are actually parallel to the *x* or *y* axis.

A simple yet original solution to such a problem is to exploit sparsity in terms of the second order mixed partial derivative. In fact, **D**_*xy*_ **χ**, that is, the vector which contains the discrete value of second mixed partial derivative, has far fewer coefficients different from zero than the gradient. For example, independently from the dimensions of the object, a rectangular scatterer will have only four elements different from zero when considering its second order mixed derivative. Then, an approach based on such a derivative can identify more easily scatterers constituted by a superposition of squares and rectangles. When such a kind of qualitative information is available, an accurate quantitative reconstruction can be obtained by solving

(6) min⁡χ Dxyχl1subject  to Aχ−bl2≤δ.

It is worth noting that the qualitative information on the morphology of the targets, which enables and suggests the use of (6), can be eventually achieved by a preliminary estimation based, for instance, on the methods in Section 3.1. Saying it in other words, procedures in Section 3.2 can be eventually seen as a possible “postprocessing” technique.

## 4. Numerical Assessment

In order to show the validity and to investigate the performances of the two proposed techniques, which aim at improving the TV-CS approach, some numerical examples with simulated data are addressed, each one dealing with a different type of scatterer.

**Figure 2:**
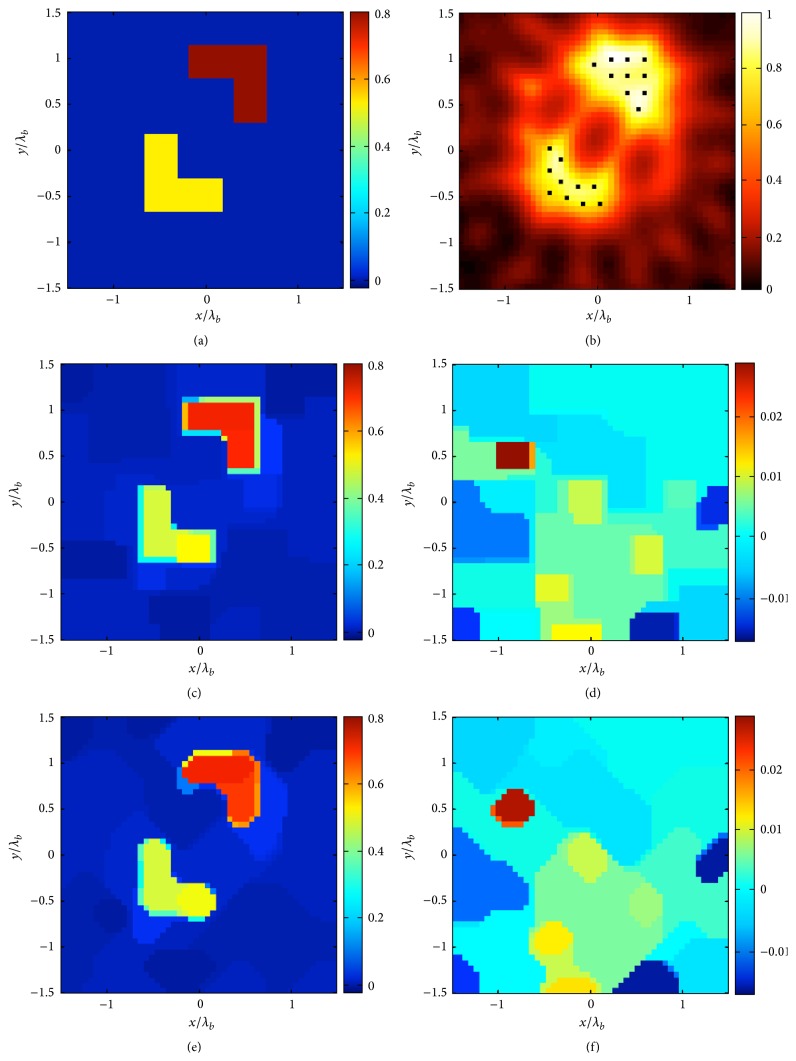
The double L scattering system. (a) Real part of the contrast reference profile. (b) Normalized logarithmic LSM indicator with the selected pivot points superimposed as dots. The retrieved profile by means of the approach (4) (*δ* = 0.2‖**b**‖_*l*_2__ and err = 9%): (c) real and (d) imaginary part. The retrieved profile by means of the approach (5) (*δ* = 0.2‖**b**‖_*l*_2__ and err = 11%): (e) real and (f) imaginary part.

**Figure 3:**
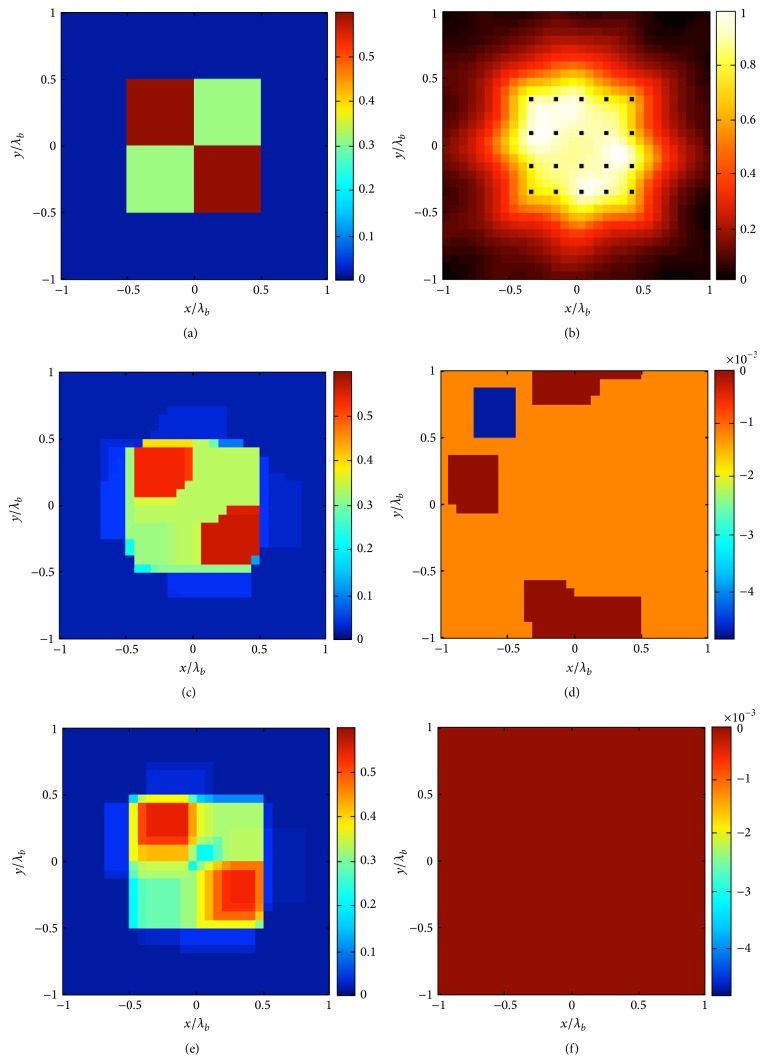
The inhomogeneous square example. (a) Real part of the contrast reference profile. (b) Normalized logarithmic LSM indicator with the selected pivot points superimposed as dots. The retrieved profile by means of the approach (4) (*δ* = 0.32‖**b**‖_*l*_2__ and err = 10%): (c) real and (d) imaginary part. The retrieved profile by means of the approach (6) (*δ* = 0.32‖**b**‖_*l*_2__ and err = 7%): (e) real and (f) imaginary part.

**Figure 4:**
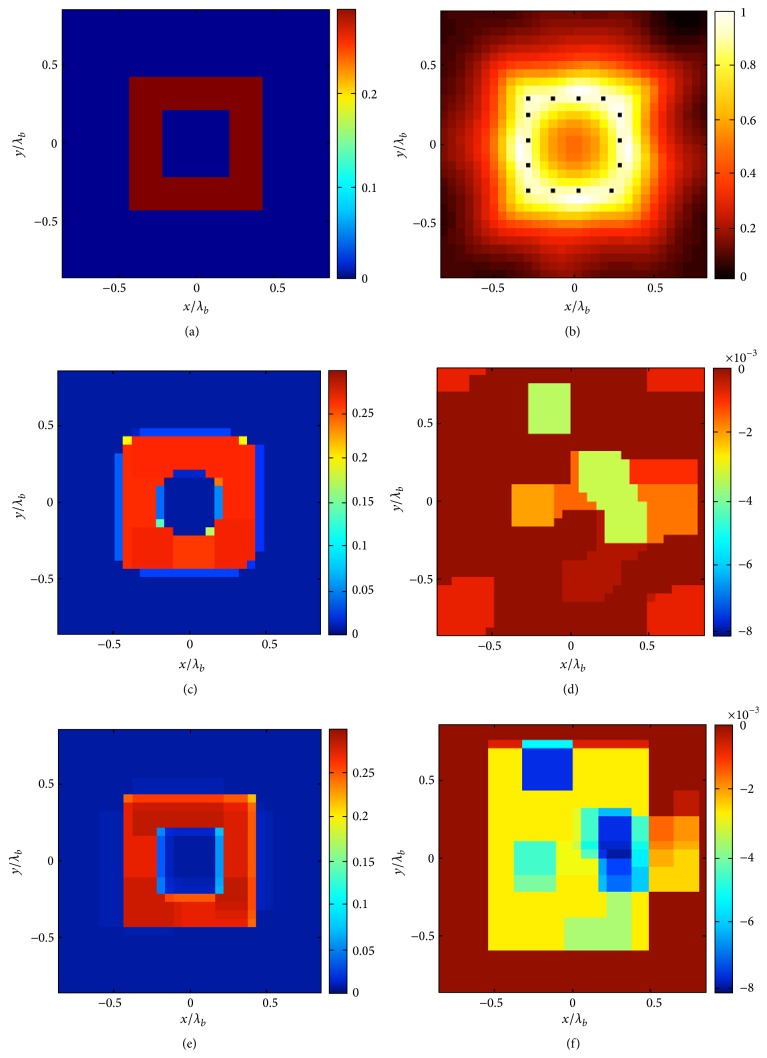
The ring square example. (a) Real part of the contrast reference profile. (b) Normalized logarithmic LSM indicator with the selected pivot points superimposed as dots. The retrieved profile by means of the approach (4) (*δ* = 0.072‖**b**‖_*l*_2__ and err = 8%): (c) real and (d) imaginary part. The retrieved profile by means of the approach (6) (*δ* = 0.072‖**b**‖_*l*_2__ and err = 1,5%): (e) real and (f) imaginary part.

In each example, we have first linearized the scattering equation, following the procedure described in Section 2 and in [9]. More in detail, the LSM equation is solved and its solution is used to build the set of virtual experiments considering a subset of pivot points inside the estimated support (see Figures 1(c), 2(b), 3(b), and 4(b)). Then, (1) is recast and linearized by using (3). At a later stage, once the problem has been linearized, the solution is looked for by means of the new introduced approaches. According to [11], in performing the numerical analysis, we set *δ* such that *δ* < ‖**b**‖_*ℓ*_2__, as a trade-off between the feasibility of the optimization task (with “feasibility” we mean the possibility to find a solution that satisfies the constraint on the data consistency) and the reconstruction accuracy. Note that if *δ* is too small, the problem could be unfeasible, as the set identified by the data constrain could be an empty set and no solution could exist at all.

Moreover, in performing the numerical analysis, the presence of the convex function *ℓ*_1_-norm in approaches (4)–(6) gives the opportunity of using the vast theory of convex optimization. In particular, the numerical examples reported in the paper have been carried out by exploiting the toolbox CVX [17, 18], a general software for convex programming.

In the following examples, the region of interest is a square of side *L*_*D*_, and the scatterer is hosted in free space. Moreover, a multiview-multistatic (MV-MS) illumination setup is assumed with filamentary currents acting as primary sources. In order to properly sample the scattered field, we consider a number *M* of measurements points equal to the minimum nonredundant number of independent scattering experiments according to [14].

The receivers and transmitters are spaced on a circumference of radius *R*. The scattered field data have been obtained by means of a full-wave forward solver based on CG-FFT procedure and corrupted with a random Gaussian noise with SNR equal to 20 dB.

For all these numerical examples, we have considered, as indicator of accuracy, the reconstruction error defined as

(7) $$\begin{array}{c} \mathrm{err}=\frac{\sum_{k=1}^{N}{\left|\chi_{k}-{\tilde{\chi }}_{k}\right|}^{2}}{\sum_{k=1}^{N}{\left|\chi_{k}\right|}^{2}}, \end{array}$$

where **χ** is the true contrast profile and $\tilde{\chi }$ is the reconstructed one.

In order to show performances of the first proposed approach, (equation (5)), in the first example we have considered a scatterer constituted by two homogeneous circular cylinders; as in the Figures 1(a) and 1(b). The dielectric permittivity of these objects is *ε* = 1.8 − 0.1798*i*. Furthermore, *N* = 48 × 48, *M* = 21, *R* = 4*λ*, and *L*_*D*_ = 1.33*λ*, where *λ* is the wavelength in the host medium. As it can be observed in Figures 1(d) and 1(e), the reconstruction obtained by using the original TV-CS approach (equation (4)) is not able to correctly identify the shape of the objects, which are reconstruced as squares rather than circles. A much more accurate solution is instead found by means of the proposed approach (see Figures 1(f) and 1(g)). In fact, the (octagonal) shape resembles more accurately the (circular) ground truth, and the reconstruction error is equal to 6%.

The second example deals with two lossless L-shape targets with different dielectric permittivity (*ε*_1_ = 1.8 and *ε*_2_ = 1.5). Furthermore, *N* = 50 × 50, *M* = 26, *R* = 4*λ*, and *L*_*D*_ = 3*λ*. As discontinuities are indeed parallel to the *x* and *y* axes, the original approach performs better than the new one (see Figure 2). However, the presence of the new term **D**_*d*_ which appears in the approach (5) still allows a satisfactory reconstruction.

In the third example, we consider an inhomogeneous square scatterer with *ε*_max⁡_ = 0.6. Furthermore, *N* = 32 × 32, *M* = 21, *R* = 4*λ*, and *L*_*D*_ = 2*λ*. In this case, we explore performances of both approaches (4) and (6). Moreover, by taking advantage of the fact that we are dealing with convex problems by virtue of the introduced approximations, we also add physical constraints on the contrast we are looking for. In particular, we enforce a positive real part and a negative imaginary part of the complex unknown **χ**. By observing Figures 3(c)–3(f), it is obvious that the new approach is able to retrieve the profile with more accuracy, as also witnessed by the reconstruction errors equal to 10% and 7% for the two cases, respectively.

In the last example, a square ring scatterer with *ε* = 1.3 is considered (see Figure 4), and we consider again both the proposed procedures (4) and (6). In particular, *N*_*c*_ = 32 × 32, *M* = 21, *R* = 4*λ*, and *L*_*D*_ = 1.33*λ*. As in the previous case, the reconstruction using the first approach already gives an accurate result and suggests that the scatterer at hand is in the class suitable for the approach described in Section 3.2. Then, application of the formulation (6) allows a still better reconstruction, achieving an error as low as 1.5%.

## 5. Conclusions

In this paper, we have introduced two new CS-TV approaches which, together with a recently introduced linear scattering model for quantitative profile inversion, allow us to achieve nearly optimal reconstructions of arbitrarily shaped and piecewise nonweak targets. In this respect, it has been shown that it is possible to improve performances of TV-CS approaches by introducing new cost functions based on directional derivatives to pursue accurate reconstructions of nonsquared objects as well as on second order derivatives to further enhance sparsity of the unknown in the case of scatterers constituted by a superposition of squares and rectangles. Joint exploitation of these concepts and their extensions to the 3D case is currently under investigation.
